# Supplementary material for: Fabricating polyoxometalates-stabilized single-atom site catalysts in confined space with enhanced activity for alkynes diboration
Source: Nat Commun. 2021 Jul 9;12:4205. doi: 10.1038/s41467-021-24513-x (PMC8271022; doi:10.1038/s41467-021-24513-x)
Supplement: Supplementary file 1 — Supplementary Information [file 41467_2021_24513_MOESM1_ESM.pdf]

Supplementary Information for

Fabricating Polyoxometalates-stabilized Single-Atom Site Catalysts in Confined Space with Enhanced Activity for Alkynes Diboration

Liu et al.

Supplementary Methods

Supplementary Figures 1-21

Supplementary Table 1-2

Supplementary References

Supplementary Methods

Synthesis of MIL-101. 1.0 g $Cr(NO_3)_3 \cdot 9H_2O$ and 0.42 g terephthalic acid were mixed in 10 mL deionized water and then stired intensely for 4 hours. The mixture was then transfered into a Teflon-lined autoclave and kept in an oven at 180 °C for 20 h without stirring. The resulting solid product was washed with distilled water and N, N-Dimethylformamide (DMF) and dried at 80 °C under ambient atmosphere for 24 hours.

Synthesis of Pt NPs@MIL-101. The synthesis of Pt NPs@MIL-101 was similar to Pt_1-PMo@MIL-101 except that there was no $H_3PMo_{12}O_{40}$ existed in the skeleton. 20 mg $Pt(acac)_2$ was dissolved in10 mL methanol and then 1.0 g MIL-101 was ultrasonic dispersed in the solution. After continuous stirring for 12 hours, the solid was collected through centrifugation and flushed with methanol to removed the surface attached $Pt(acac)_2$. The resulted product was dried at 80 °C in vacuum oven for 24 hours, and then reduced in 5% H_2 at 150 °C for 1 hour to obtain Pt NPs@MIL-101. The Pt content was determined by ICP-OES with about 0.24 wt%.

Synthesis of Pt NPs@PMo. 20 mg $Pt(acac)_2$ and 1g $H_3PMo_{12}O_{40}$ were dissolved in 50 mL ethanol. After continuous stirring for 4 hours, the solvent was evaporated. The powder was reduced in 5% H_2 at 150 °C for 1 hour and then washed with ethanol and water to remove the excess $H_3PMo_{12}O_{40}$. The resulted product was dried at 80 °C in vacuum oven for 24 hour to obtain Pt NPs@PMo.

Syntheses of Directing Agent Solution. NaOH, $NaAlO_2$, and H_2O were mixed and stirred until the solution is clear. Then 40 wt% colloidal silica was dropwise added to the solution, followed by a continuous stirring for 4 hours. Then the mixture was aged for 3 days under room temperature. The final molar composition of the directing agent is 18.4 Na_2O: 1.0 Al_2O_3: 18.5 SiO_2: 366 H_2O.

Syntheses of Y zeolite. NaOH, $NaAlO_2$, and H_2O were mixed and stirred until the solution is clear. Then 40 wt% colloidal silica and directing agent (20 wt% of the starting gel) was dropwise added to the solution consecutively. After a continuous stirring for 4 hours and aged for 4 hours at room temperture. The reaction mixture was then transferred into a Teflon-lined stainless steel autoclave and the crystallization was conducted in a conventional oven at 100 °C for 12 hours. The final molar composition of the starting gel is 3.36 Na_2O: 1.0 Al_2O_3: 8.4 SiO_2: 250 H_2O. The as-synthesized solid products were centrifuged, washed with water and ethanol for several times, and then dried at 80 °C in the vacuum oven overnight.

Synthesis of Pt NPs@Y zeolite. Pt NPs@Y was synthesized by the approaches reported previously with some modification.[1] Platinum was introduced as the tetra-ammine ion complex from queous solutions of the chloride. The Y zeolite was used as support. The obtained catalyst was washed with water followed by reduction in H_2 (5%) at 600 ^oC for 1 hour.

Synthesis of Pt_1@Y zeolite. $PtCl_2$ and excess ethylenediamine (EDA) were mixed in distilled water and stirred until the solution is clear, then NaOH, $NaAlO_2$, SiO_2, and directing agent (20 wt% of the starting gel) were added to the aqueous solution successively. The final molar composition of the starting gel is 3.36 Na_2O: 1.0 Al_2O_3: 8.4 SiO_2: 250 H_2O: 0.015 $PtCl_2$: 2.0 EDA, after a continuous stirring for 4 hours and aged for 4 hours at room temperture. The reaction mixture was then transferred into a Teflon-lined stainless steel autoclave and the crystallization was conducted in a conventional oven at 100 °C for 12 hours. The as-synthesized solid products were centrifuged, washed with water and ethanol for several times to remove excess Pt-EDA complex, and then dried at 80 °C in the vacuum oven overnight. Then the obtained Pt-EDA@Y was calcined in static air at 400 °C for 2 hours and reduced in flowing H_2 (5%) at 200 °C for 1 hours to prepare Pt_1@Y.

Computational methods: All density functional theory (DFT) calculations were performed by Vienna Ab intio Simulation Package (VASP)[2,3]. The projected augmented wave (PAW) potential[4,5] and generalized gradient approximation of Perdew-Burke-Ernzerhof (PBE) functional[6,7] were employed to describe the electron-ion interaction and exchange-correlation energy, respectively. The DFT-D3 method[8] was employed to correct the van der Waals interaction. During structure relaxation, the energy cutoff was set to 400 eV for the plane-wave expansion, the energy convergence was set to 10^{-5} eV and all the structures were fully relaxed until the residual force on each atom was smaller than 0.01 eV/Å. To obtain the accurate electronic structure, the cutoff energy was increased to 520 eV when calculating density of states of Pt single atom (Pt-SA). To simulate the coordination environment of Pt-SA, the Pt was stabilized by 4 surficial oxygen atoms of Keggin molecule, which was placed in a vacuum cage of 30×30×30 Å to avoid the interaction between the replicas due to the periodic boundary condition. The Pt(111) surface was modeled by 8×8 supercell with a vacuum layer larger than 20 Å and three atomic layers, the bottom layer of which was fixed.

Supplementary Figures

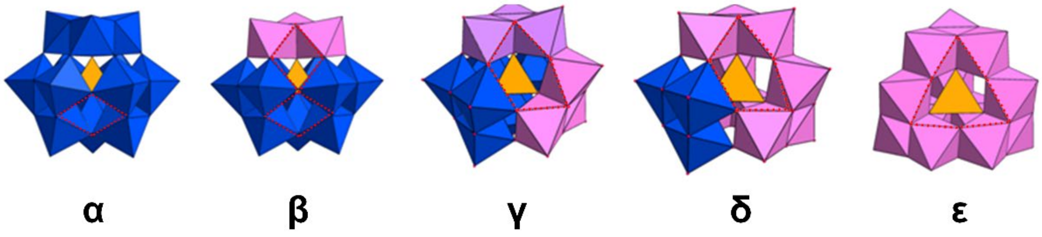

Supplementary Fig. 1 Five types of isomers of Keggin-type POMs with different anchoring sites for single metal atoms.

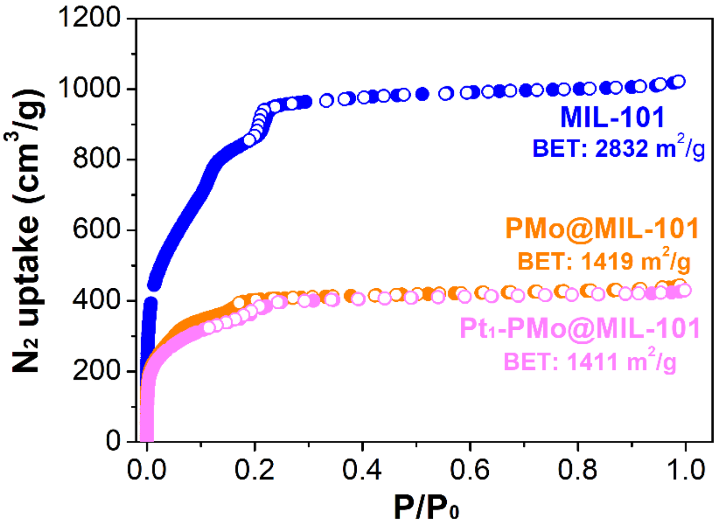

Supplementary Fig. 2 N_2 adsorption/desorption isotherms of MIL-101, PMo@MIL-101, and Pt_1-PMo@MIL-101 respectively.

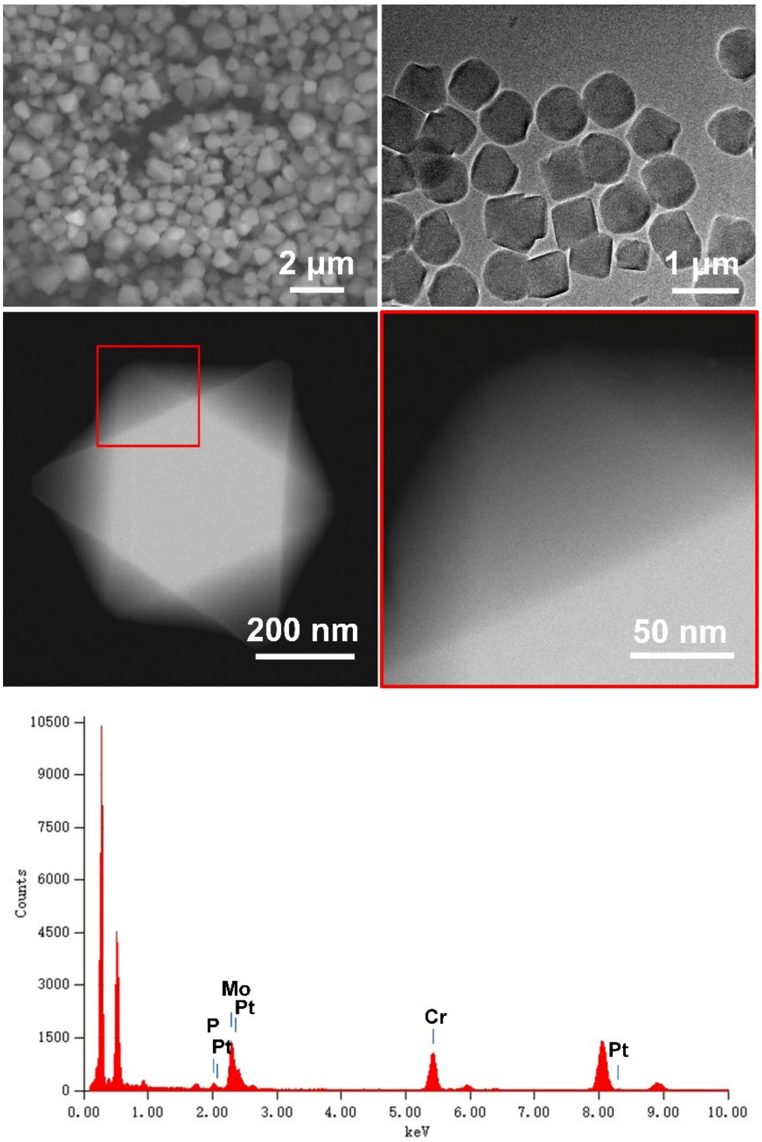

Supplementary Fig. 3 SEM, TEM, and HAADF-STEM images of Pt_1-PMo@MIL-101, and EDS spectra of Pt_1-PMo@MIL-101 correxponding to Figure 3b.

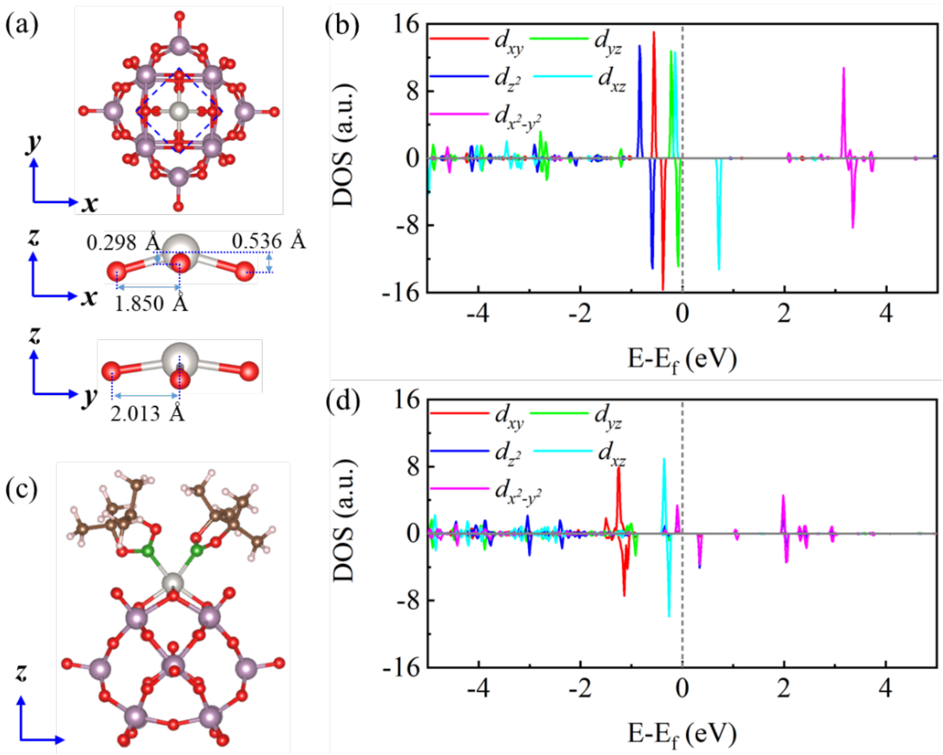

Supplementary Fig. 4 (a) Structure of Pt_1-PMo@MIL-101 and magnification of Pt_1 coordination environment (blue dashed square). (b) PDOS of *d*-orbital of single Pt atom in PMo@MIL-101. (c) Structure of Pt_1-PMo@MIL-101 after adsorption of $Bpin_2$ molecule. (d) PDOS of *d*-orbital of single Pt atom after adsorption.

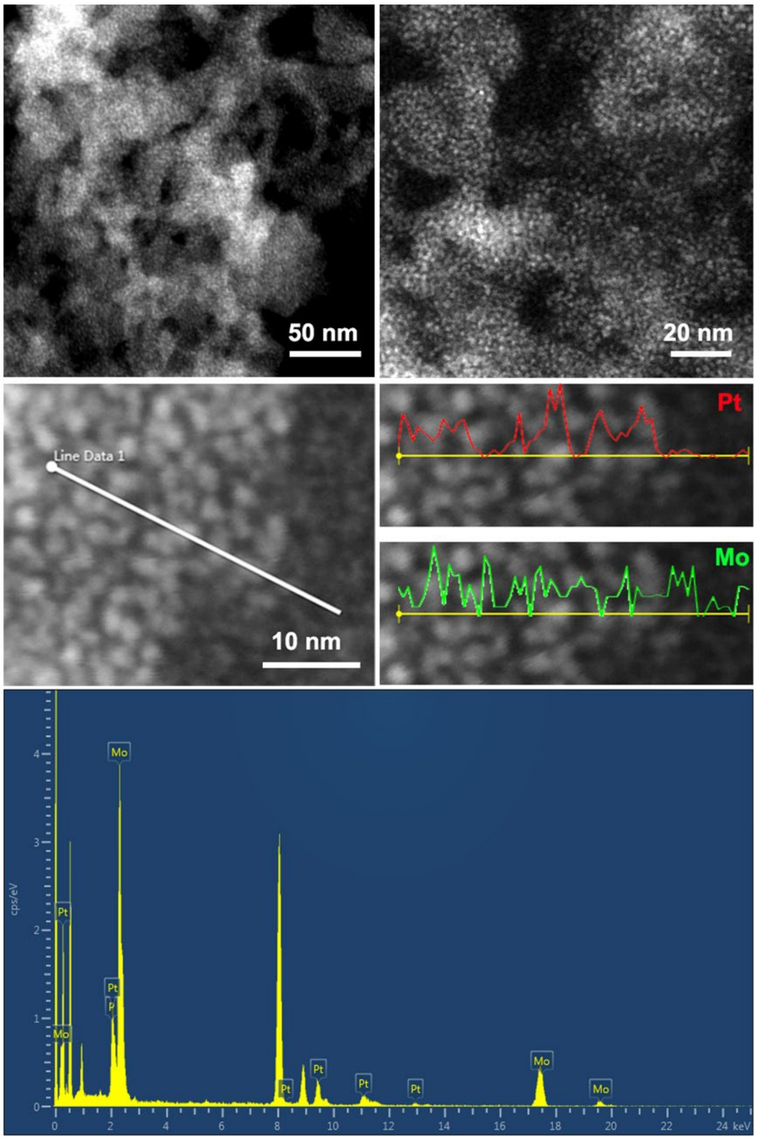

Supplementary Fig. 5 HAADF-STEM images and corresponding element line scan and EDS spectra of Pt NPs@PMo.

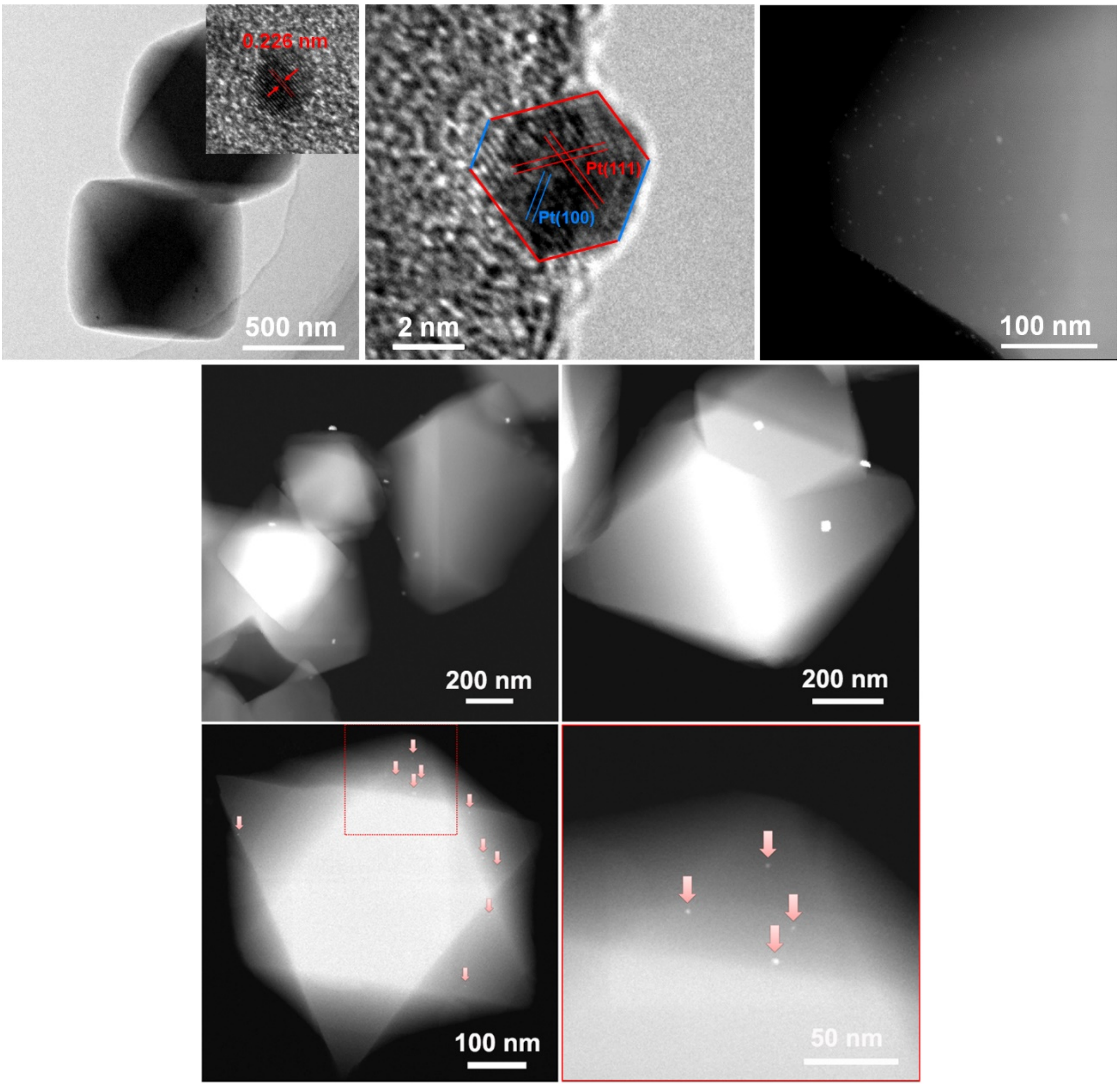

Supplementary Fig. 6 TEM, HRTEM, and HAADF-STEM images of Pt NPs@MIL-101 without PMo. The measured (111) lattice fringe spacing is 0.226 nm.

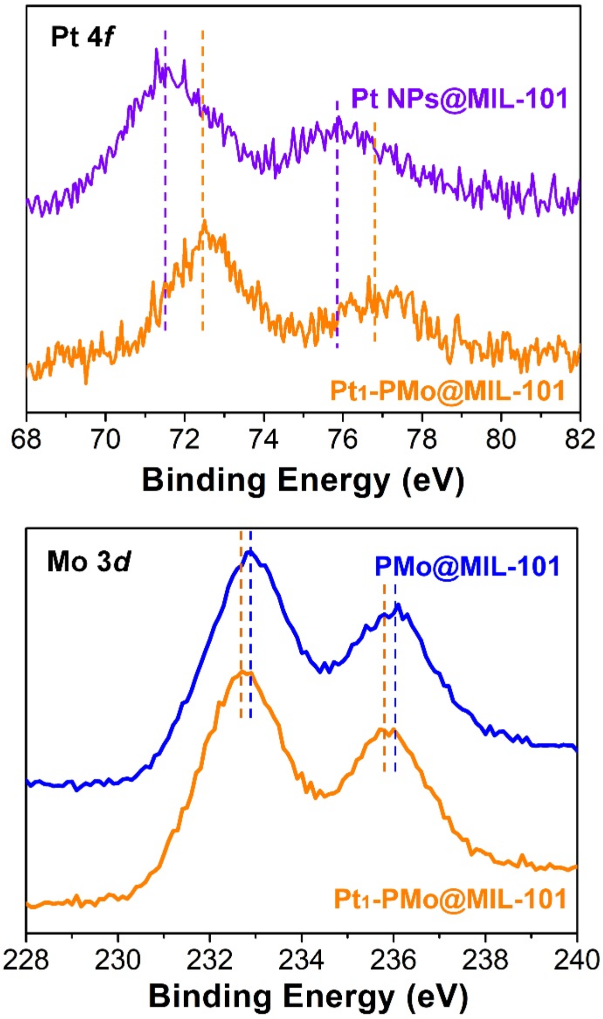

Supplementary Fig. 7 Pt 4*f* XPS spectra of Pt NPs@MIL-101 and Pt_1-PMo@MIL-101. Mo 3*d* XPS spectra of PMo@MIL-101 and Pt_1-PMo@MIL-101.

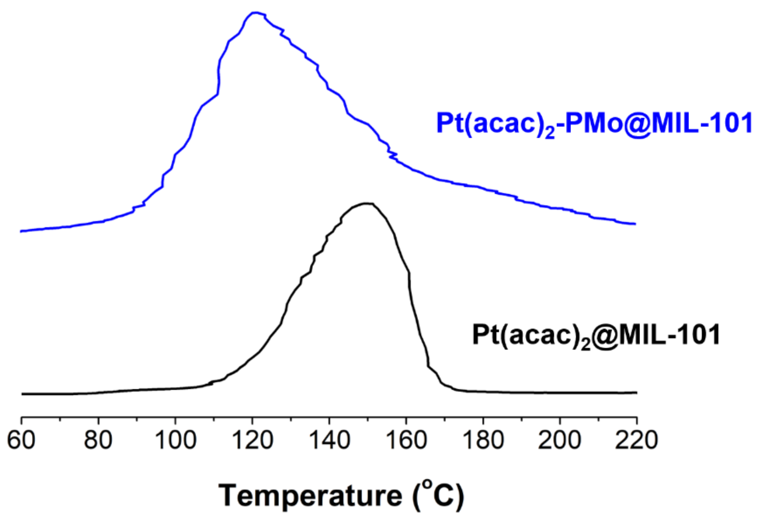

Supplementary Fig. 8 H_2-TPR profiles of Pt(acac)$_2$@MIL-101 and Pt(acac)$_2$-PMo@MIL-101.

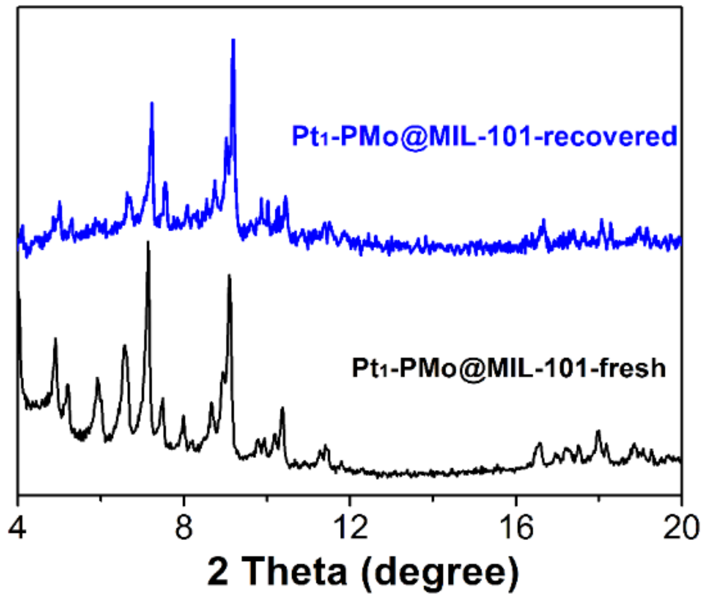

Supplementary Fig. 9 PXRD of fresh Pt_1-PMo@MIL-101and recovered Pt_1-PMo@MIL-101 after five cycles.

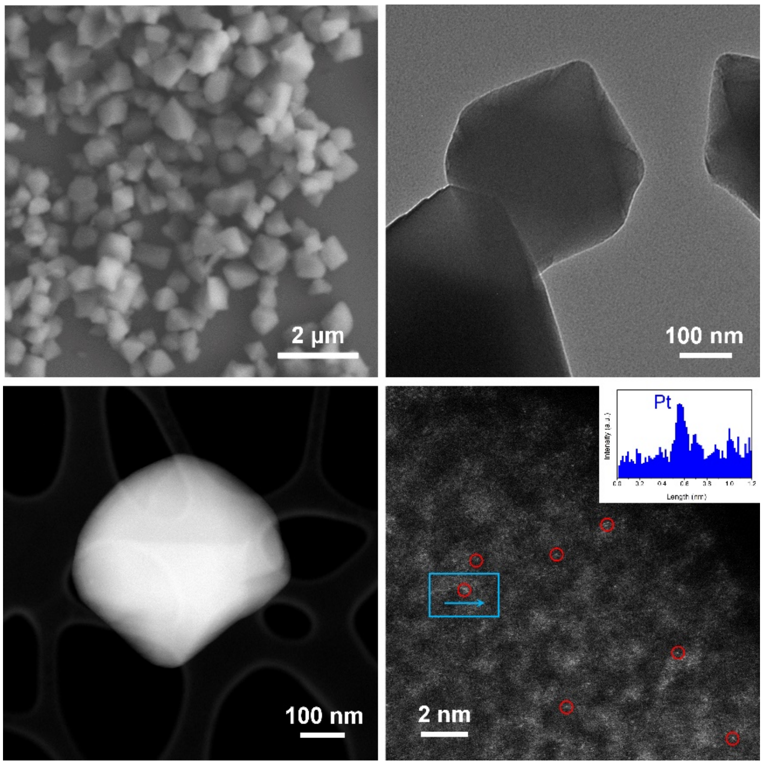

Supplementary Fig. 10 SEM, TEM, HAADF-STEM, Aberration-corrected HAADF STEM images and corresponding Z-contrast analysis of recovered Pt_1-PMo@MIL-101.

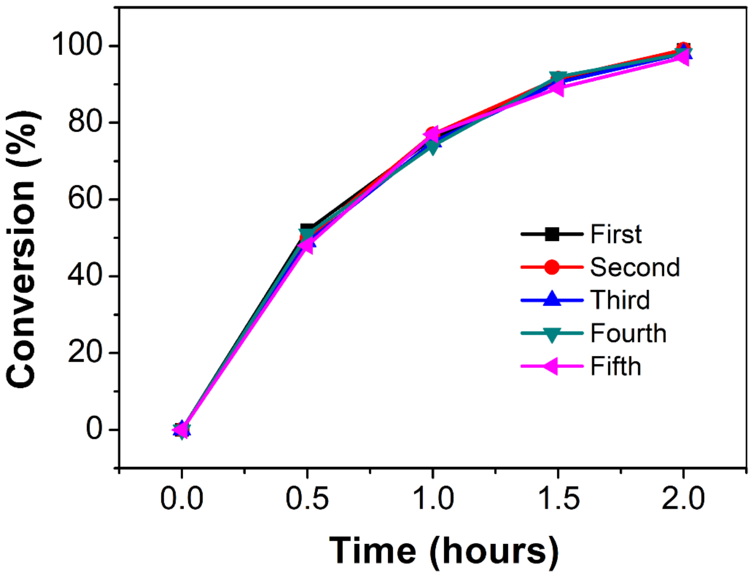

Supplementary Fig. 11 The temporal catalytic performance of recovered Pt_1-PMo@MIL-101 in different cycles.

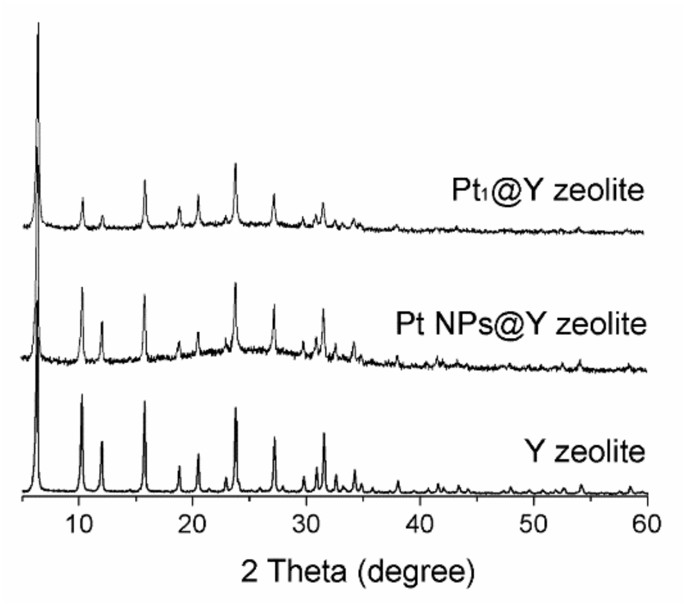

Supplementary Fig. 12 PXRD of Pt_1@Y zeolite and Pt NPs@Y zeolite.

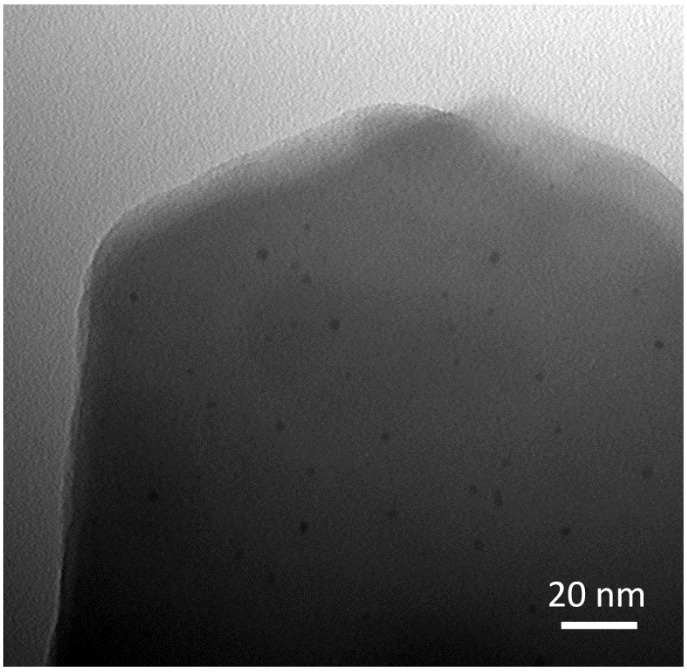

Supplementary Fig. 13 TEM image of Pt NPs@Y zeolite.

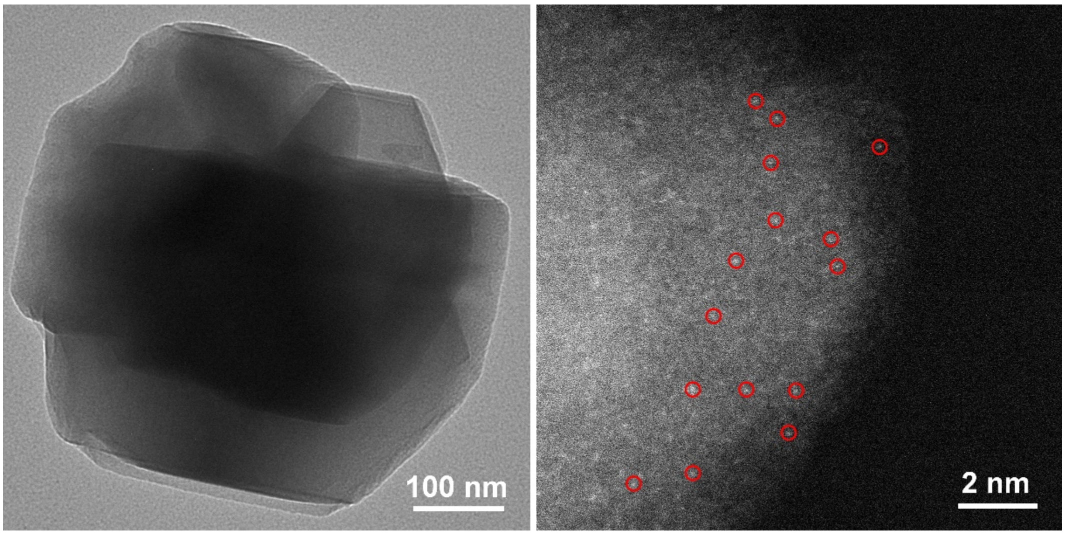

Supplementary Fig. 14 TEM and Aberration-corrected HAADF STEM images of Pt_1@Y zeolite.

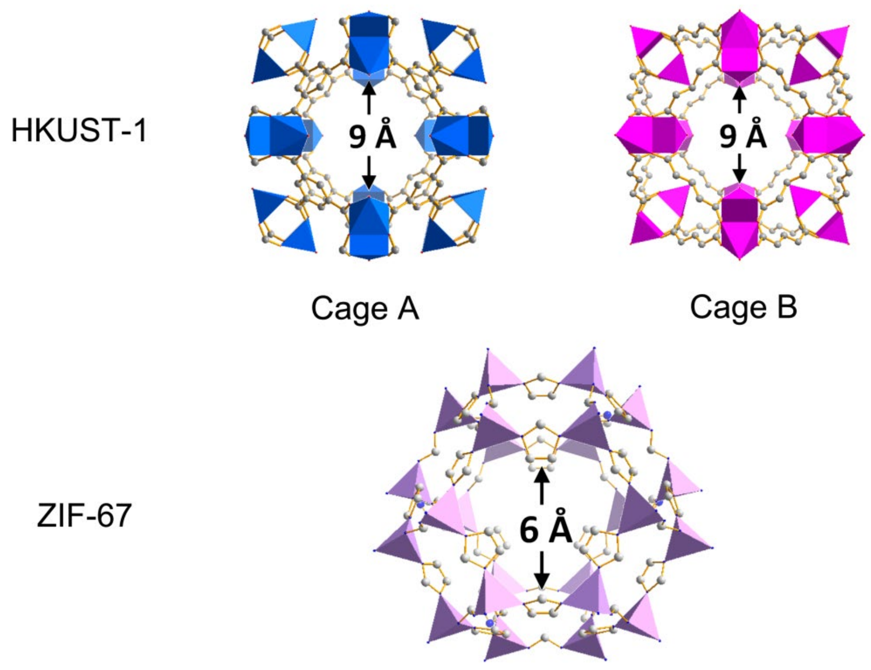

Supplementary Fig. 15 The pore structures and windows diameters of HKUST-1 and ZIF-67.

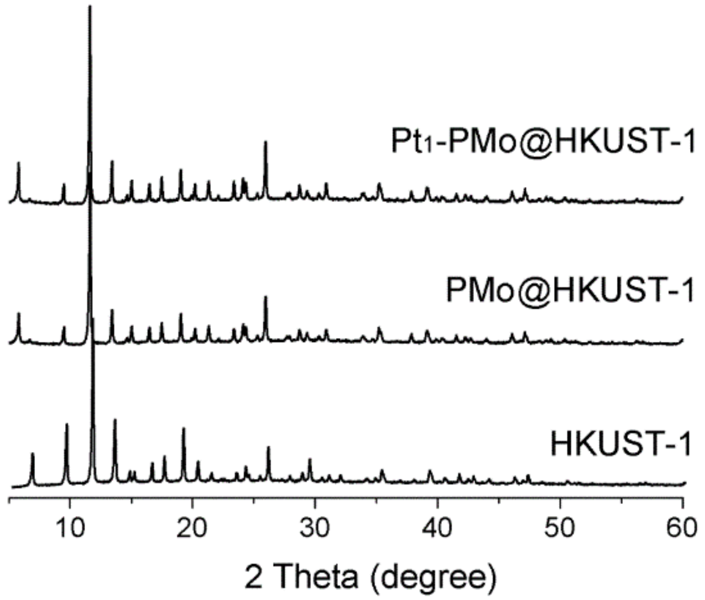

Supplementary Fig. 16 PXRD of HKUST-1, PMo@HKUST-1, and Pt_1-PMo@HKUST-1 respectively.

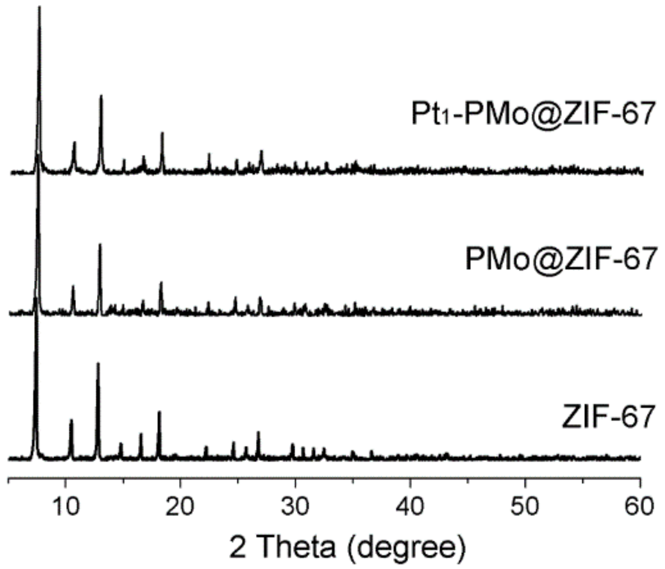

Supplementary Fig. 17 PXRD of ZIF-67, PMo@ZIF-67, and Pt_1-PMo@ZIF-67 respectively.

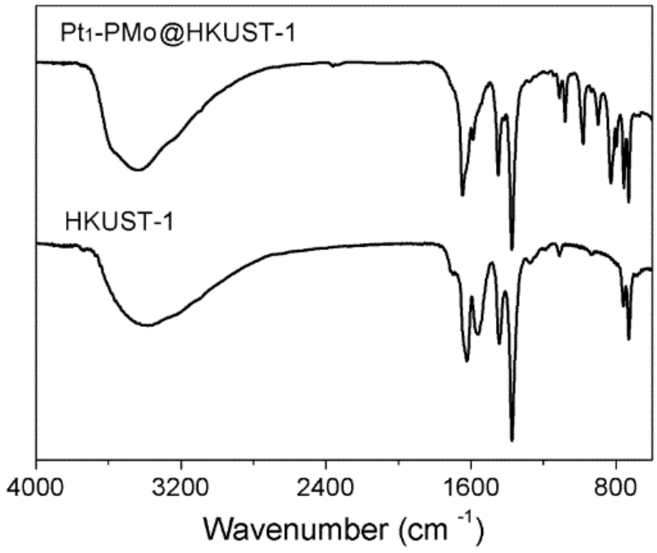

Supplementary Fig. 18 FTIR of HKUST-1 and Pt_1-PMo@HKUST-1 respectively.

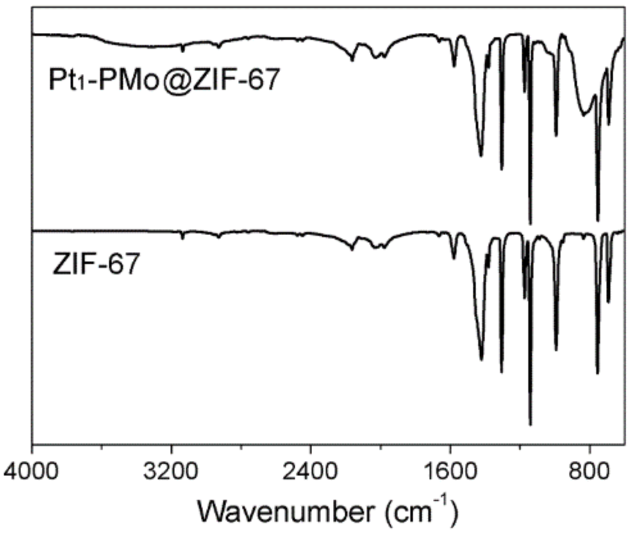

Supplementary Fig. 19 FTIR of ZIF-67 and Pt_1-PMo@ZIF-67 respectively.

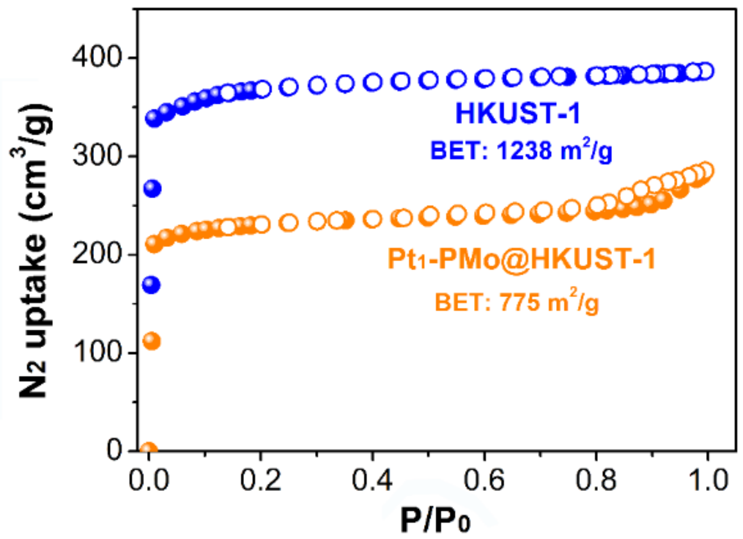

Supplementary Fig. 20 N_2 adsorption/desorption isotherms of HKUST-1 and Pt_1-PMo@HKUST-1 respectively.

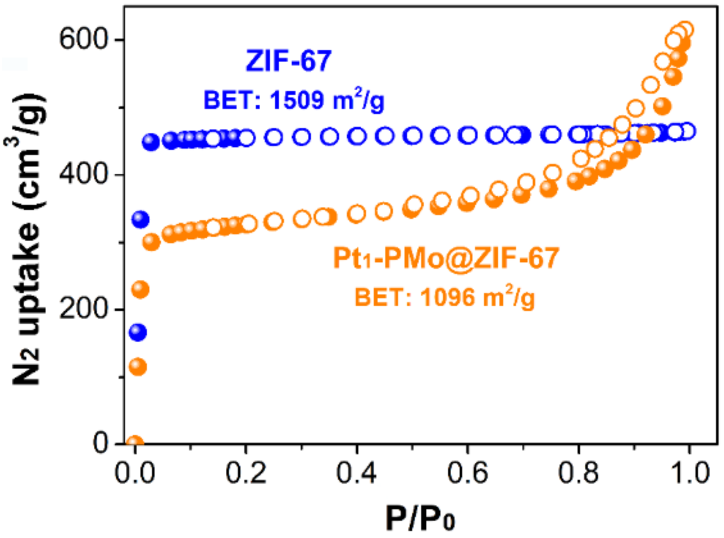

Supplementary Fig. 21 N_2 adsorption/desorption isotherms of ZIF-67 and Pt_1-PMo@ZIF-67 respectively.

Supplementary Table 1. Structural parameters of Pt_1-PMo@MIL-101 extracted from the EXAFS fitting. (S_0^2=0.85)

Scattering pair	CN	R (Å)	σ^2 (10^{-3}Å^2)	ΔE_0 (eV)	R factor
Pt -O	4.3±0.6	1.97±0.02	5.6±0.7	-4.6±0.4	0.01

S_0^2 is the amplitude reduction factor; CN is the coordination number; R is interatomic distance (the bond length between central atoms and surrounding coordination atoms); σ^2 is Debye-Waller factor (a measure of thermal and static disorder in absorber-scatterer distances); ΔE_0 is edge-energy shift (the difference between the zero kinetic energy value of the sample and that of the theoretical model). R factor is used to value the goodness of the fitting.

Supplementary Table 2. The formation energy (E_f) of three different Pt anchoring sites on PMo surface.

Site	E_{tot}	E_{sub}	E_{Pt}	E_f
4O	-445.6699	-437.2388	-6.8616	-1.5695
3O-1	-443.6323	-437.2388	-6.8616	0.4681
3O-2	-443.7377	-437.2388	-6.8616	0.3627

In order to determin the most stable anchoring site of Pt atom on the surface of PMo, we introduced formation energy (E_f) which is defined as:

$$E_f = E_{tot} - E_{sub} - E_{Pt} \qquad (1)$$

where E_{tot} is the total energy of substrate adsorbed with Pt atom, E_{sub} is the energy of substrate without adsorbate, E_{Pt} is the energy of per Pt atom in the bulk structure.

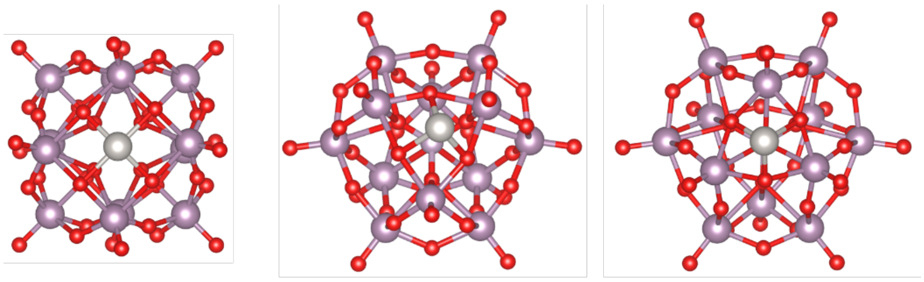

4O 3O-1 3O-2

Supplementary References

1. L. Xu, *et al.* Mechanistic study of preferential CO oxidation on a Pt/NaY zeolite catalyst. *J Catal* **287**, 114-123 (2012).

2. Kresse G, Furthmüller J. Efficiency of ab-initio total energy calculations for metals and semiconductors using a plane-wave basis set. *Comp Mater Sci* **6**, 15-50 (1996).

3. Kresse G, Furthmüller J. Efficient iterative schemes for ab initio total-energy calculations using a plane-wave basis set. *Phys Rev B* **54**, 11169 (1996).

4. Blöchl P E. Projector augmented-wave method. *Phys Rev B* **50**, 17953 (1994).

5. Kresse G, Joubert D. From ultrasoft pseudopotentials to the projector augmented-wave method. *Phys Rev B* **59**, 1758 (1999).

6. Perdew J P, Burke K, Ernzerhof M. Generalized gradient approximation made simple. *Phys Rev Lett* **77**, 3865 (1996).

7. Perdew J P, Ernzerhof M, Burke K. Rationale for mixing exact exchange with density functional approximations. *J Chem Phys* **105**, 9982-9985 (1996).

8. Grimme S. Semiempirical GGA-type density functional constructed with a long-range dispersion correction. *J Comp Chem* **27**, 1787-1799 (2006).
